# Supplementary material for: Isolation and Structure Determination of New Pyrones from Dictyostelium spp. Cellular Slime Molds Coincubated with Pseudomonas spp
Source: Molecules. 2024 May 5;29(9):2143. doi: 10.3390/molecules29092143 (PMC11085369; doi:10.3390/molecules29092143)
Supplement: Supplementary file 1 [file molecules-29-02143-s001.zip › molecules-2988940-supplementary.pdf]

Supporting Information

Isolation and Structure Determination of New Pyrones from *Dictyostelium* spp. Cellular Slime Molds Coincubated with *Pseudomonas* spp.

Takehiro Nishimura, Takuya Murotani, Hitomi Sasaki, Yoshinori Uekusa, Hiromi Eguchi, Yuzuru Kubohara, and Haruhisa Kikuchi

Table of contents

	page
^{1}H NMR spectrum ($CDCl_3$, 600 MHz) for clavapyrone (**1**) (Figure S1)	S2
^{13}C NMR and DEPT spectrum ($CDCl_3$, 150 MHz) for clavapyrone (**1**) (Figure S2)	S3
DQF-COSY spectrum ($CDCl_3$, 600 MHz) for clavapyrone (**1**) (Figure S3)	S4
HMQC spectrum ($CDCl_3$, 600 MHz) for clavapyrone (**1**) (Figure S4)	S5
HMBC spectrum ($CDCl_3$, 600 MHz) for clavapyrone (**1**) (Figure S5)	S6
^{1}H NMR spectrum ($CDCl_3$, 600 MHz) for intermedipyrone (**2**) (Figure S6)	S7
^{13}C NMR and DEPT spectrum ($CDCl_3$, 150 MHz) for intermedipyrone (**2**) (Figure S7)	S8
DQF-COSY spectrum ($CDCl_3$, 600 MHz) for intermedipyrone (**2**) (Figure S8)	S9
HMQC spectrum ($CDCl_3$, 600 MHz) for intermedipyrone (**2**) (Figure S9)	S10
HMBC spectrum ($CDCl_3$, 600 MHz) for intermedipyrone (**2**) (Figure S10)	S11
Experimental and theoretical ECD spectrum for intermedipyrone (**2**) (Figure S11)	S12
Structures and specific rotation data of isocoumarines (Table S1)	S12
^{1}H NMR spectrum ($CDCl_3$, 600 MHz) for magnumiol (**3**) (Figure S12)	S13
^{13}C NMR and DEPT spectrum ($CDCl_3$, 150 MHz) for magnumiol (**3**) (Figure S13)	S14
DQF-COSY spectrum ($CDCl_3$, 600 MHz) for magnumiol (**3**) (Figure S14)	S15
HMQC spectrum ($CDCl_3$, 600 MHz) for magnumiol (**3**) (Figure S15)	S16
HMBC spectrum ($CDCl_3$, 600 MHz) for magnumiol (**3**) (Figure S16)	S17
HRMS spectra for clavapyrone (**1**) (Figure S17)	S18
HRMS spectra for intermedipyrone (**2**) (Figure S18)	S18
HRMS spectra for magnumiol (**3**) (Figure S19)	S19
Reference	S20

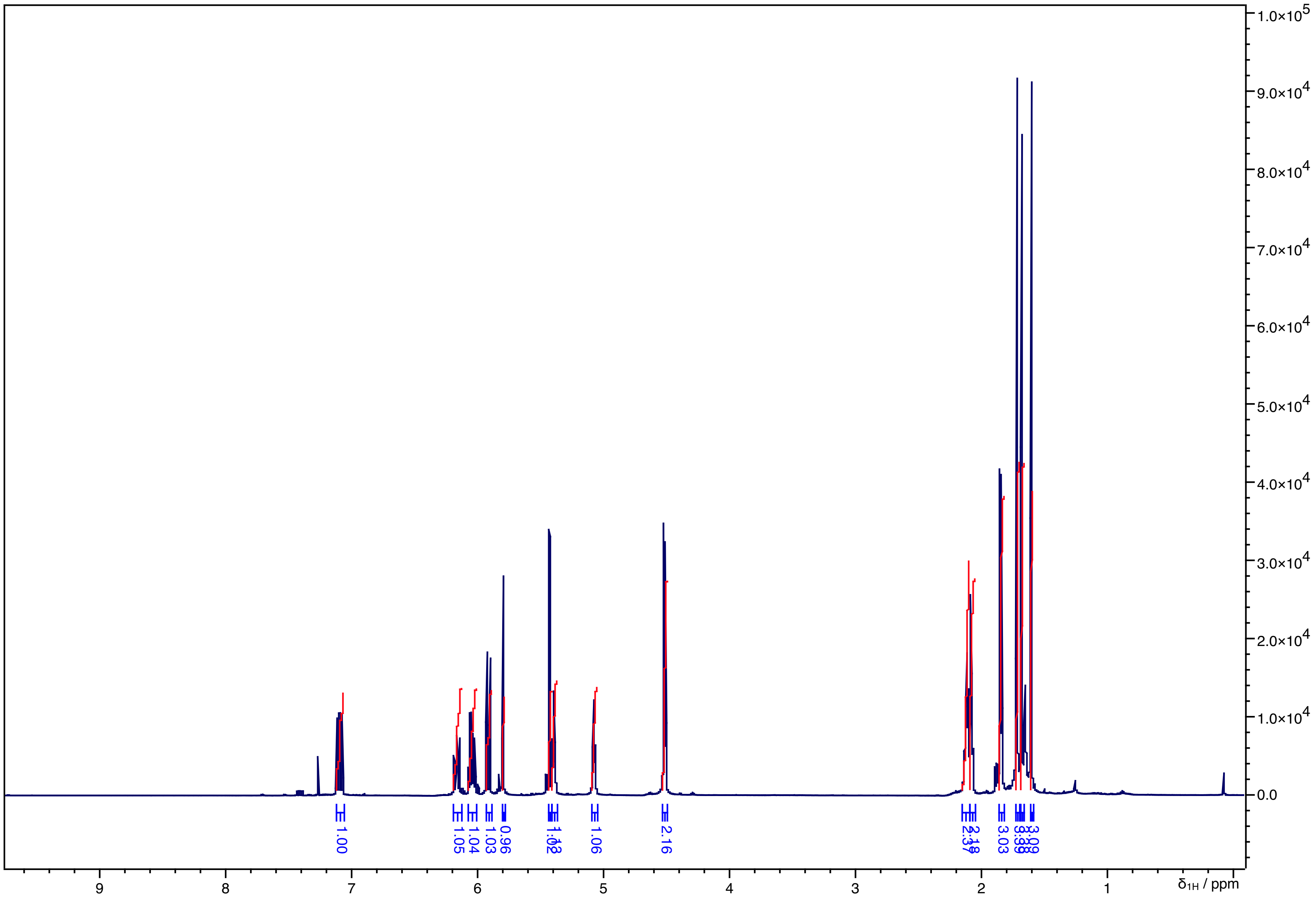

Figure S1. ^{1}H NMR spectrum ($CDCl_3$, 600 MHz) for **1**.

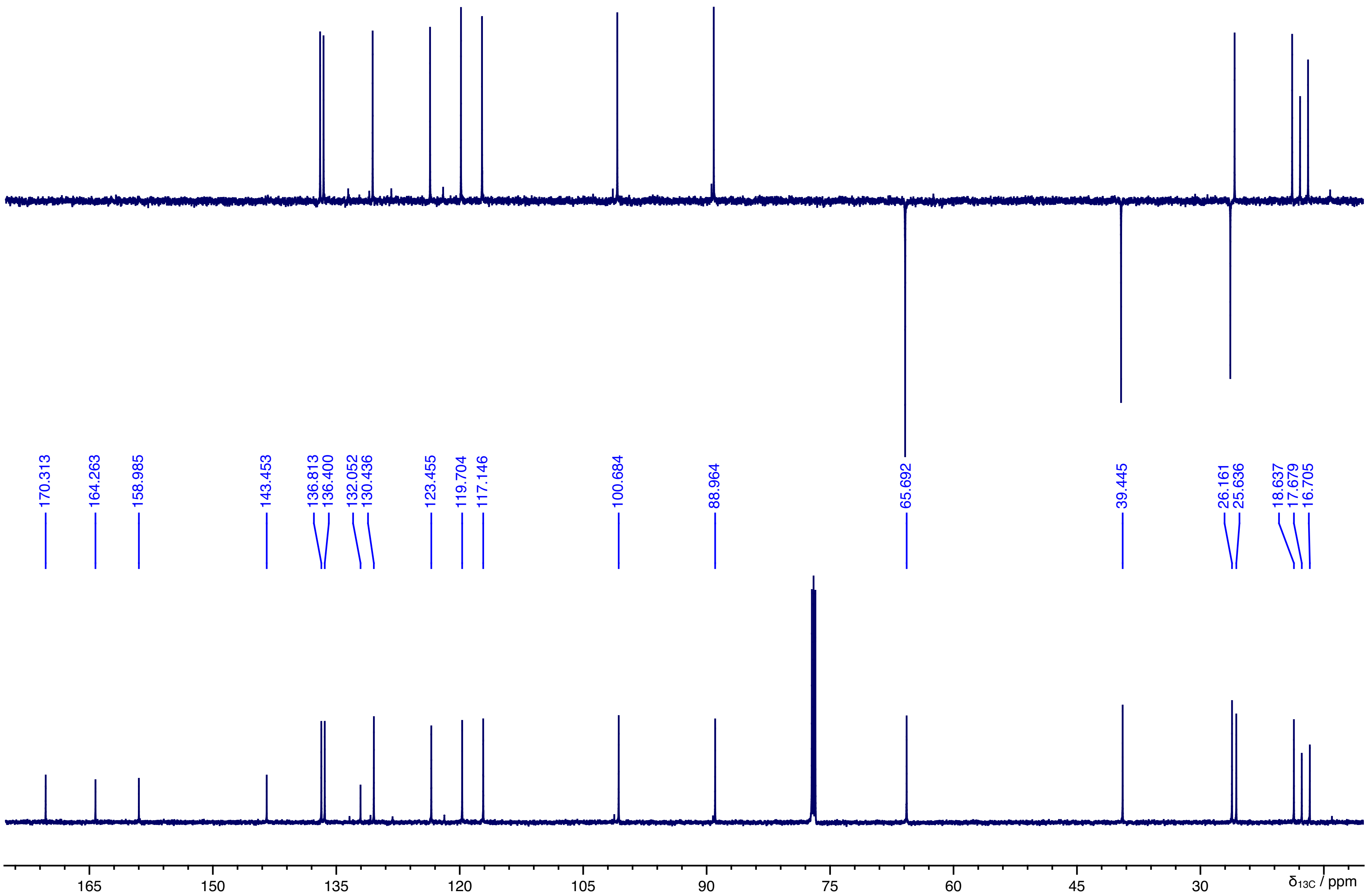

Figure S2. ^{13}C NMR and DEPT spectra ($CDCl_3$, 150 MHz) for **1**.

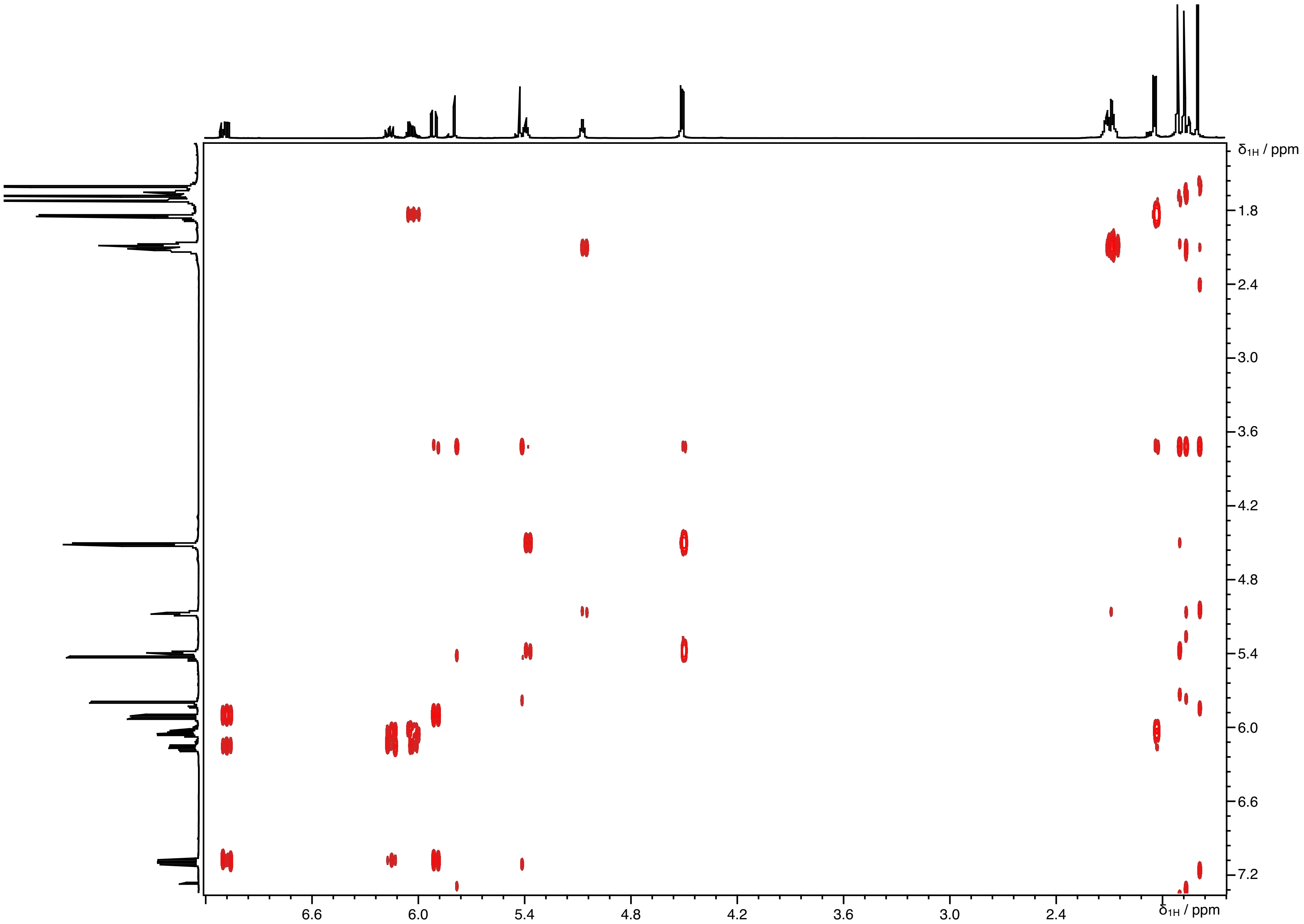

Figure S3. DQF-COSY spectrum ($CDCl_3$, 600 MHz) for **1**.

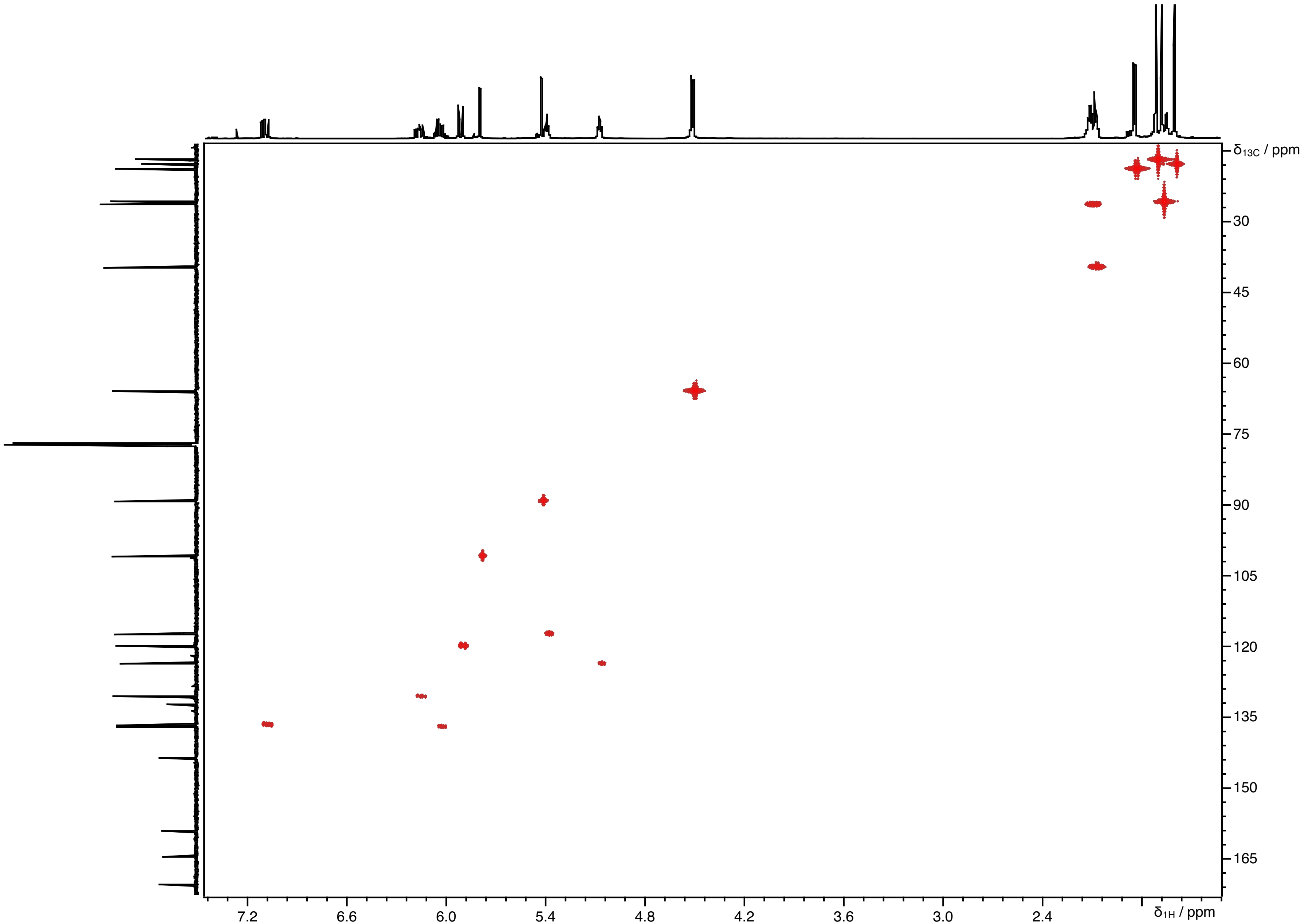

Figure S4. HMQC spectrum ($CDCl_3$, 600 MHz) for **1**.

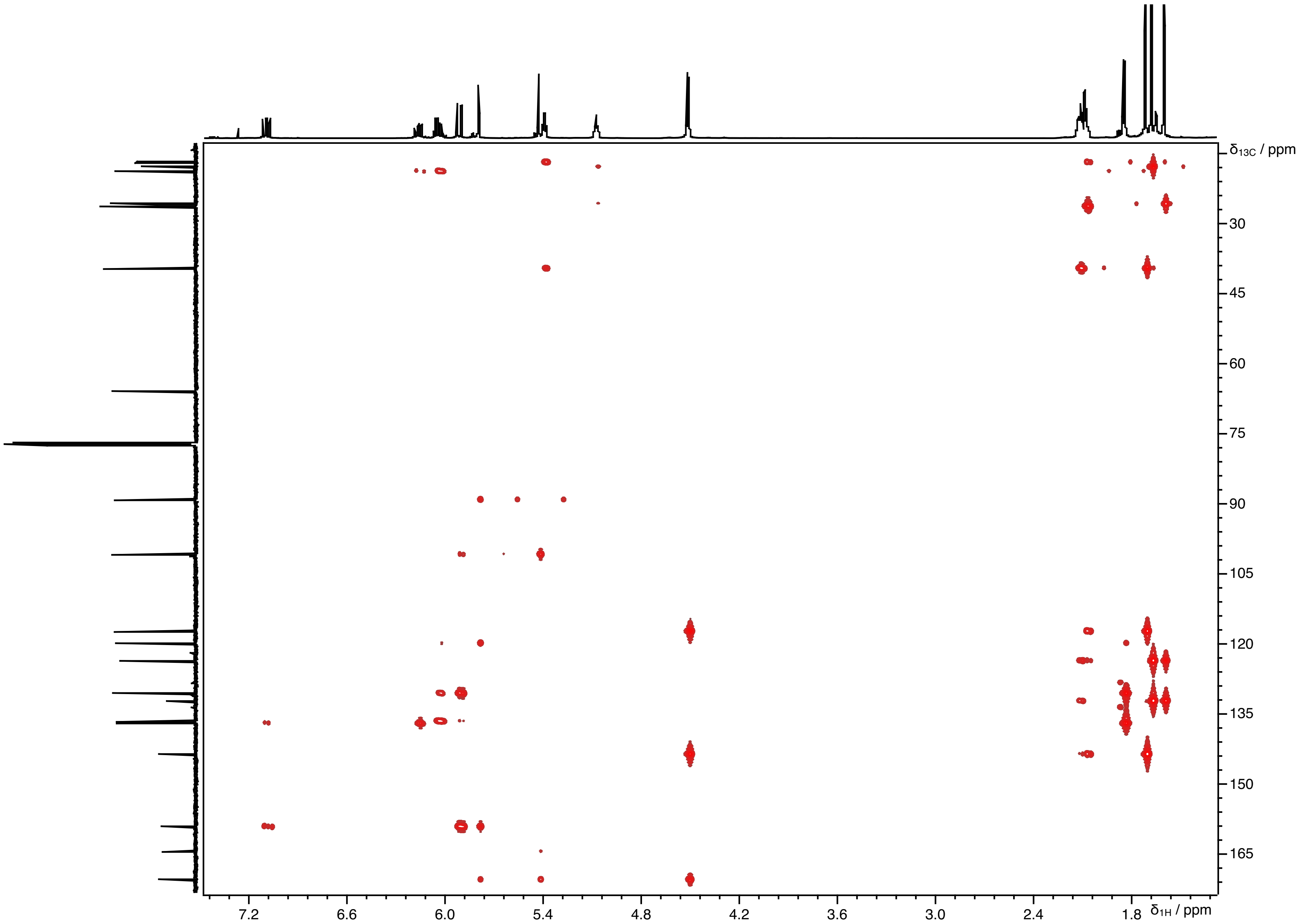

Figure S5. HMBC spectrum ($CDCl_3$, 600 MHz) for **1**.

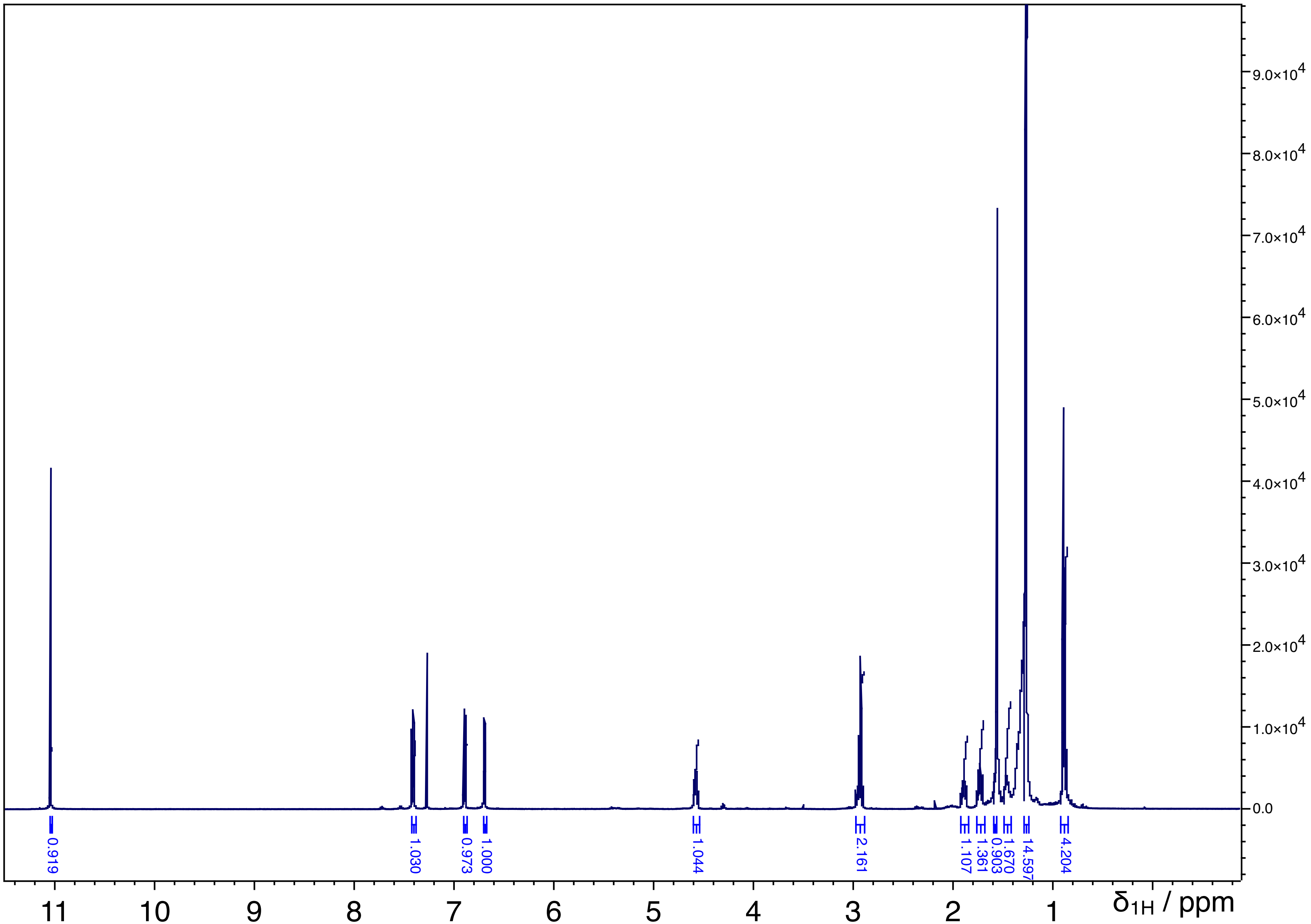

Figure S6. ^{1}H NMR spectrum ($CDCl_3$, 600 MHz) for **2**.

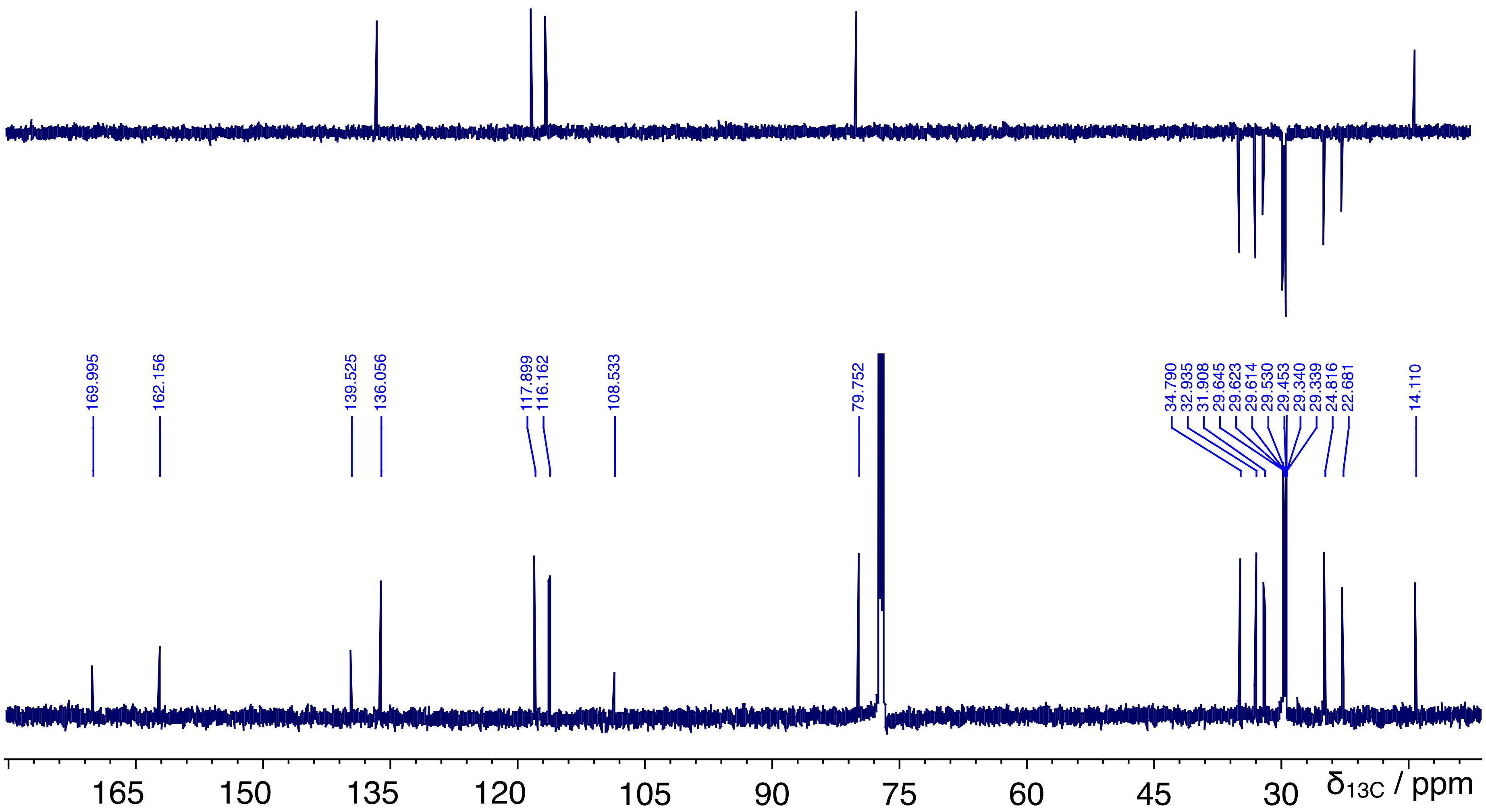

Figure S7. ^{13}C NMR and DEPT spectra ($CDCl_3$, 150 MHz) for **2**.

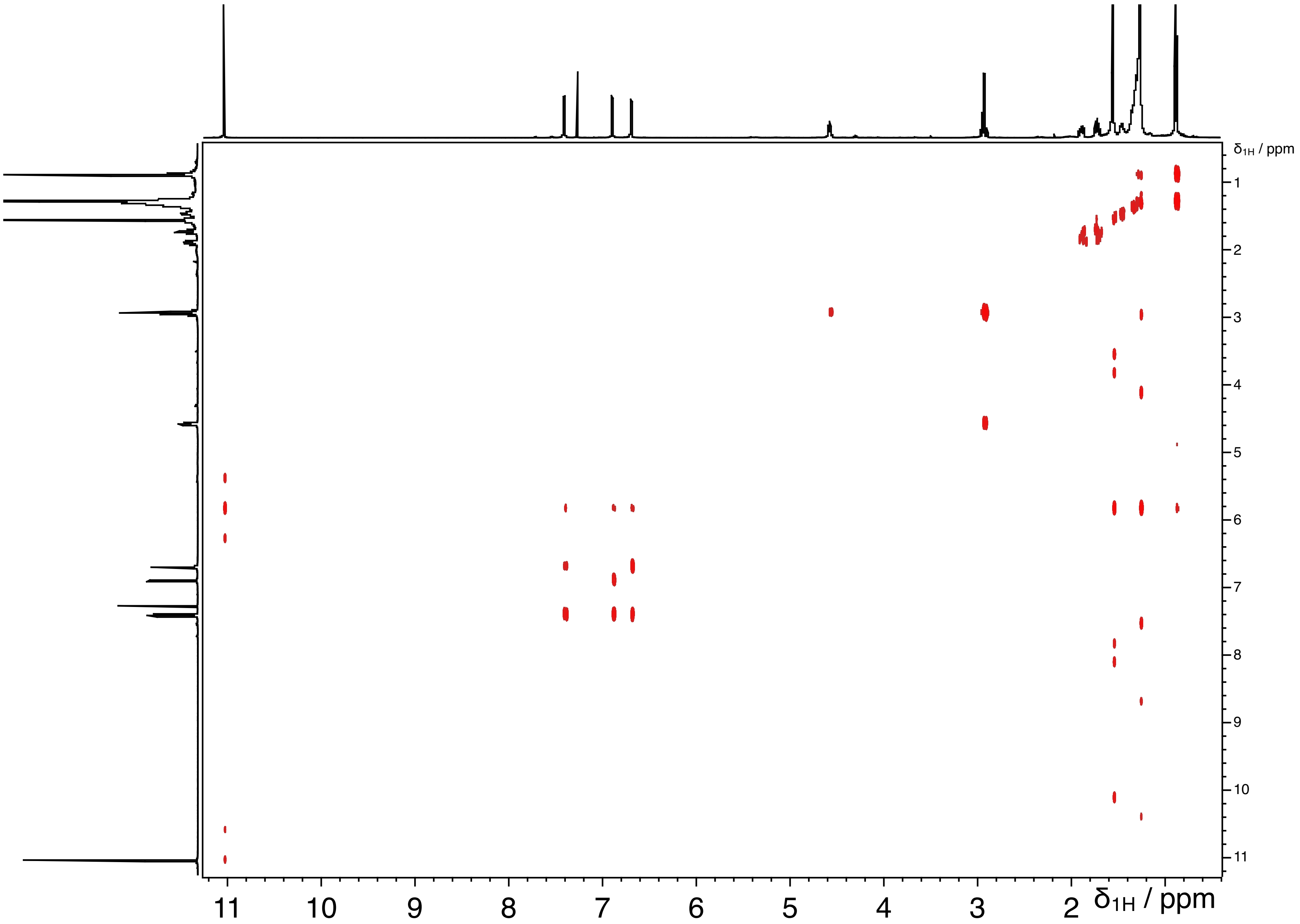

Figure S8. DQF-COSY spectrum ($CDCl_3$, 600 MHz) for **2**.

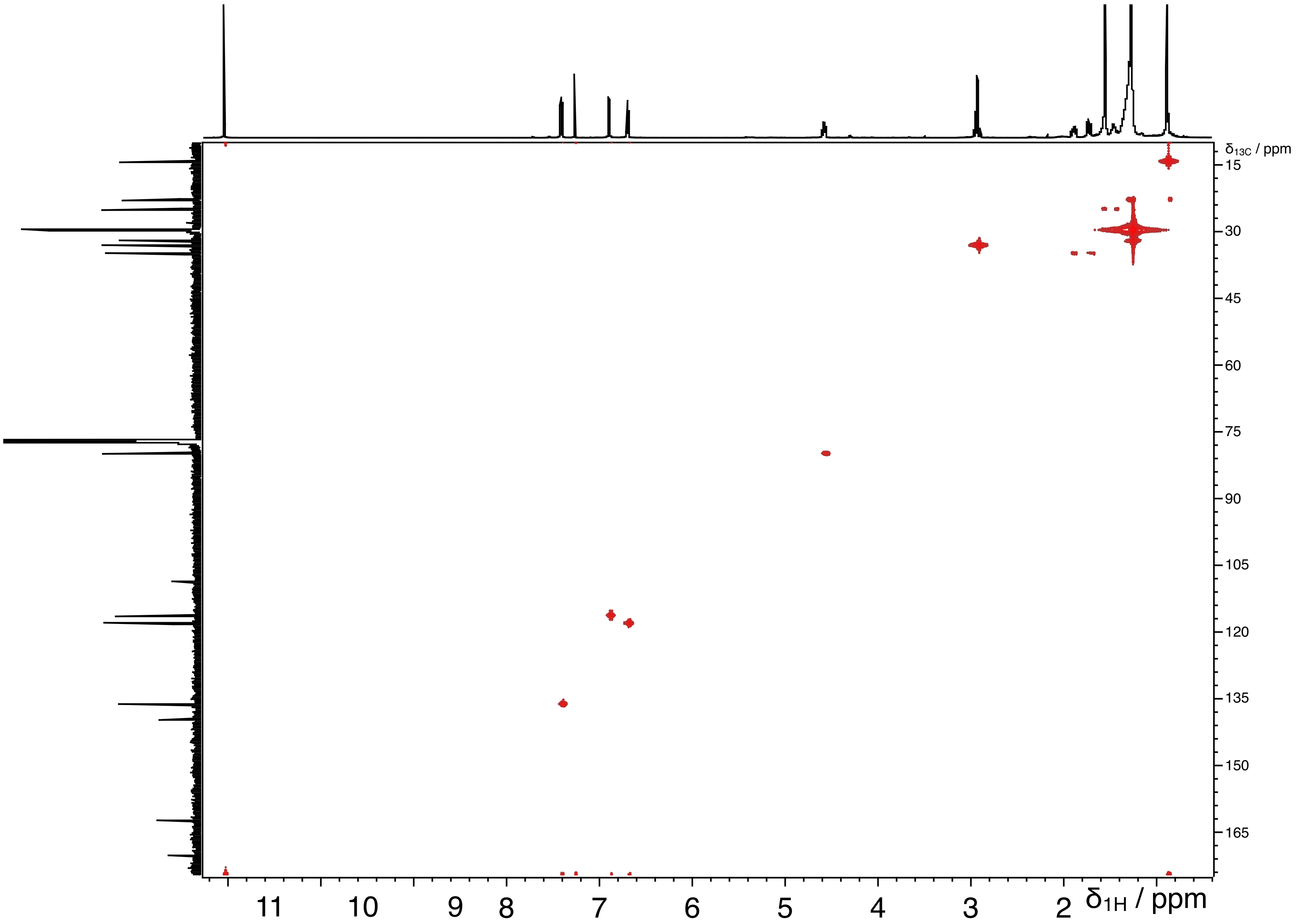

Figure S9. HMQC spectrum ($CDCl_3$, 600 MHz) for **2**.

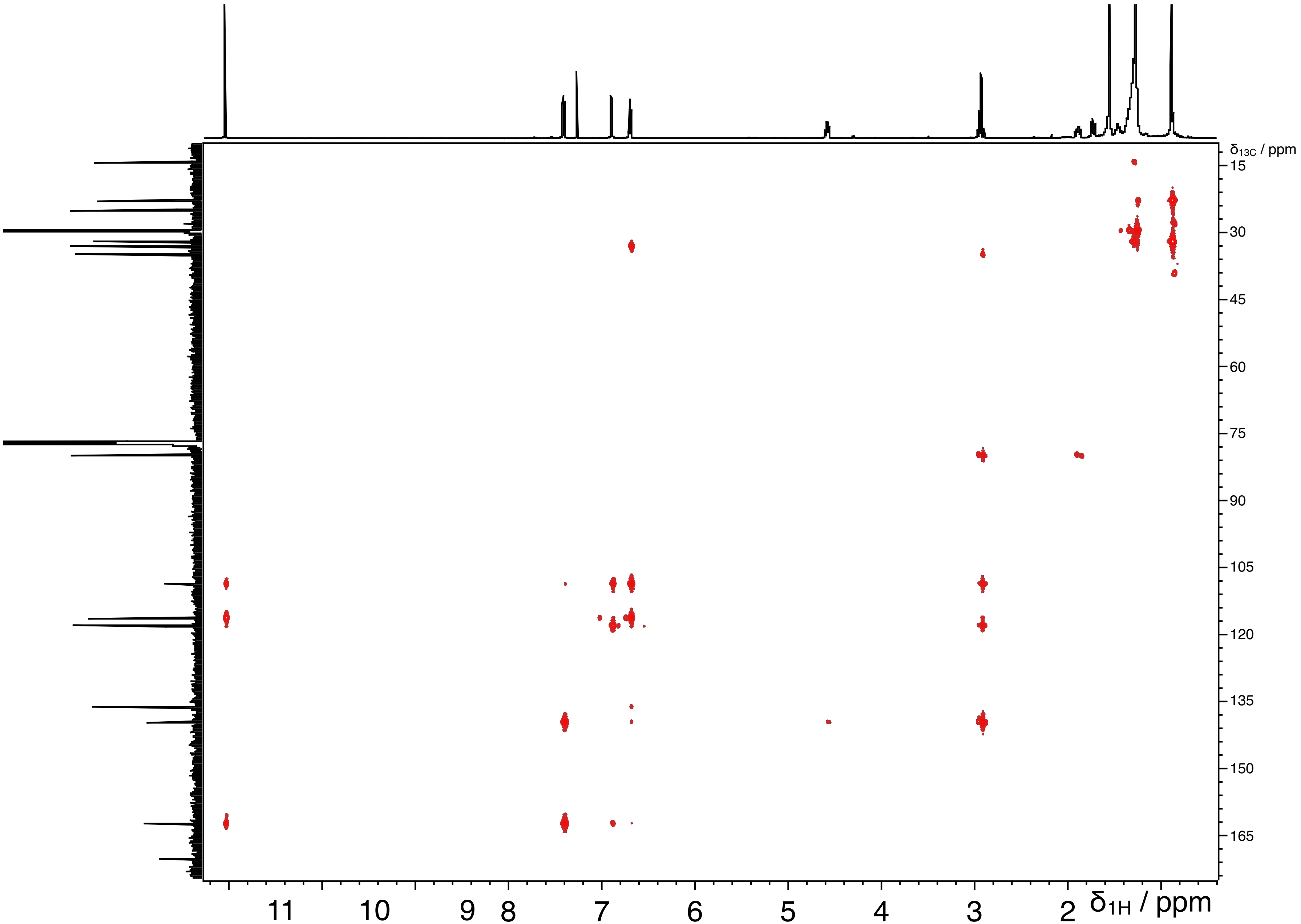

Figure S10. HMBC spectrum ($CDCl_3$, 600 MHz) for **2**.

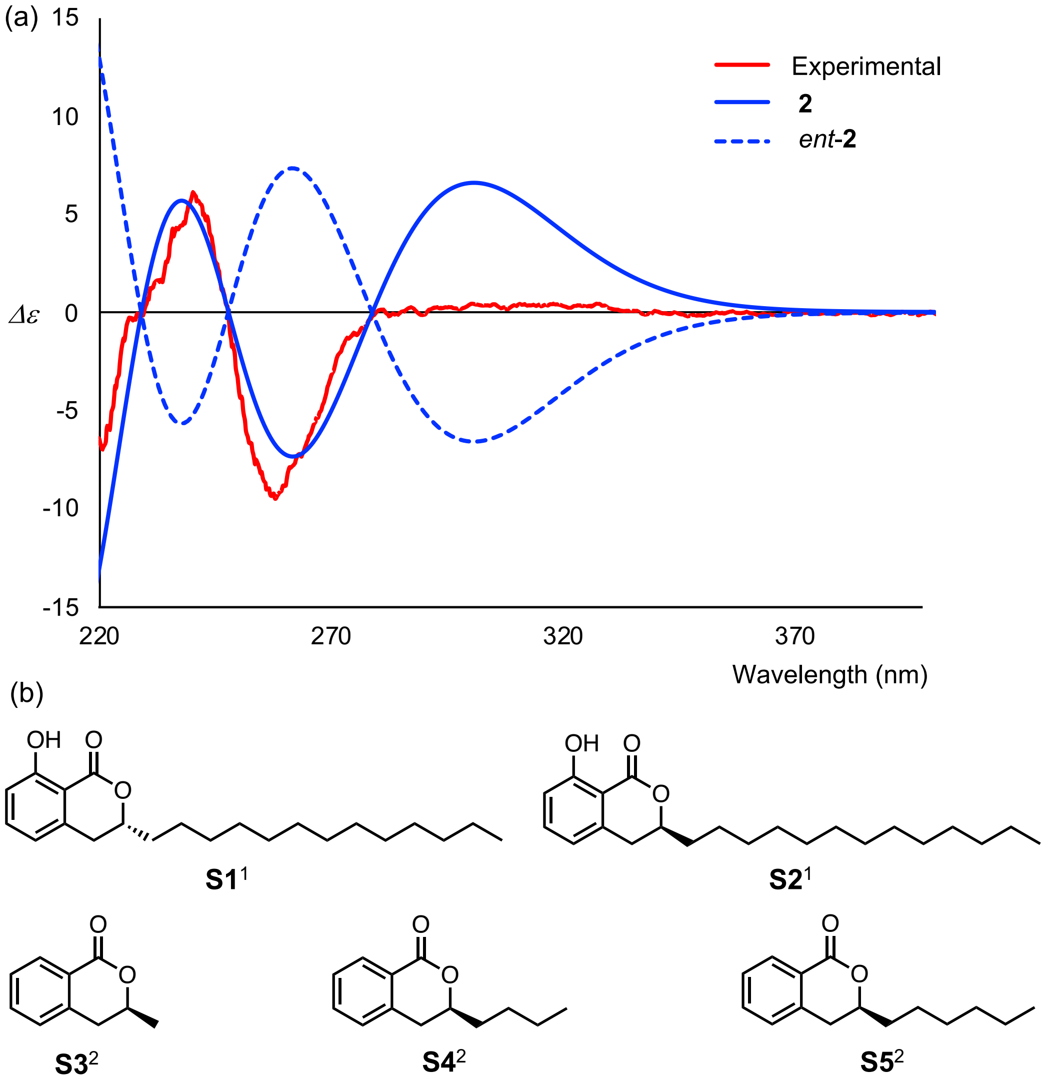

Figure S11. (a) Experimental and theoretical ECD spectra of **2** and *ent*-**2**. (b) Structures used for comparison of ECD spectra and optical rotations with **2**.

Table S1. Specific rotations of isocoumarin-type compounds.[1,2]

Compounds	Specific rotation
**2**	$[\alpha]^{26}_{D}$ –20.4 (c 0.15, $CDCl_3$)
**S1**	$[\alpha]^{23}_{D}$ –31 (c 1.0, $CDCl_3$)
**S2**	$[\alpha]^{23}_{D}$ +32 (c 1.0, $CDCl_3$)
**S3**	$[\alpha]^{22}_{D}$ +141.4 (c 1.30, $CDCl_3$)
**S4**	$[\alpha]^{22}_{D}$ +82.9 (c 1.20, $CDCl_3$)
**S5**	$[\alpha]^{22}_{D}$ +68.9 (c 1.25, $CDCl_3$)

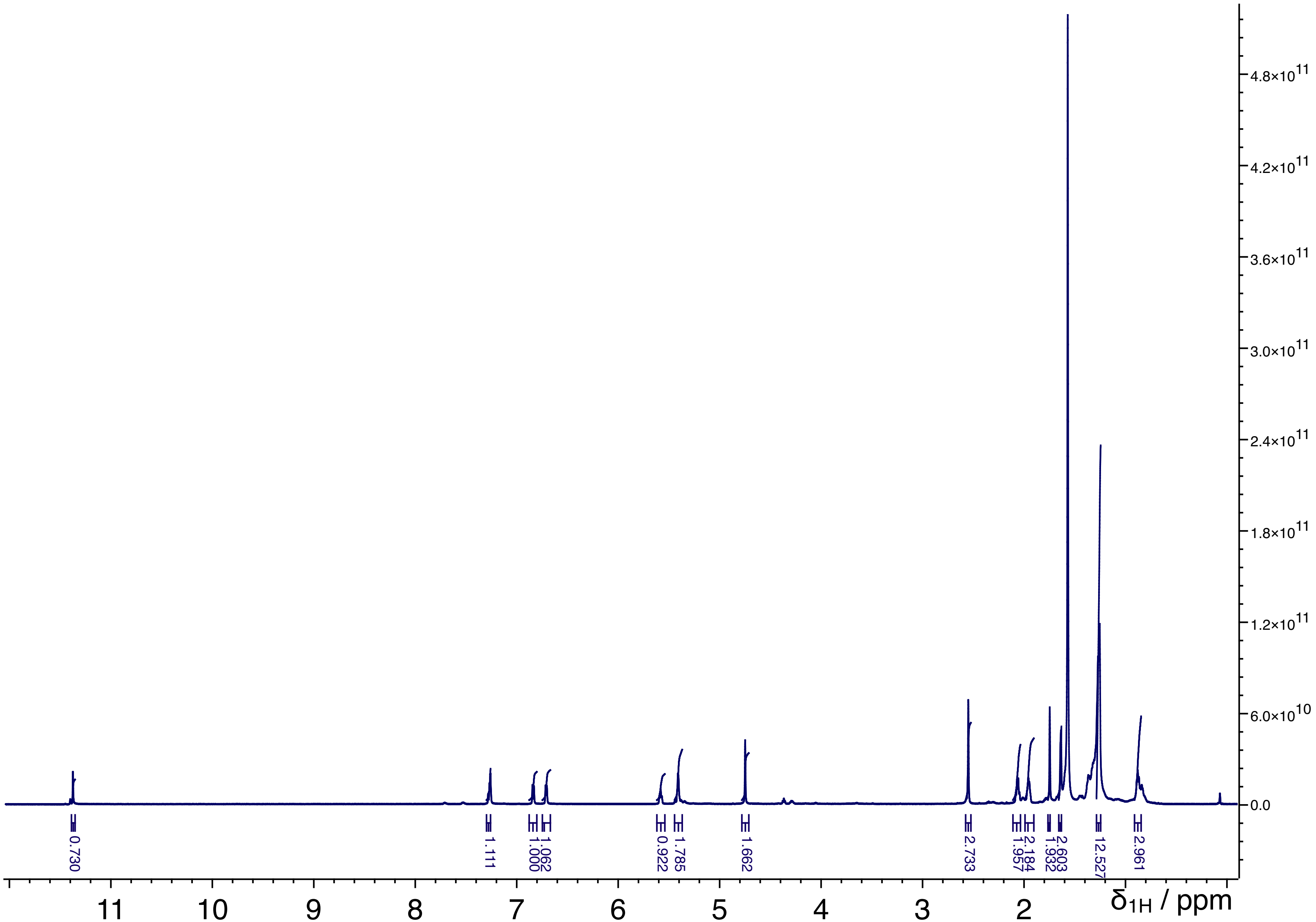

Figure S12. ^{1}H NMR spectrum ($CDCl_3$, 600 MHz) for **3**.

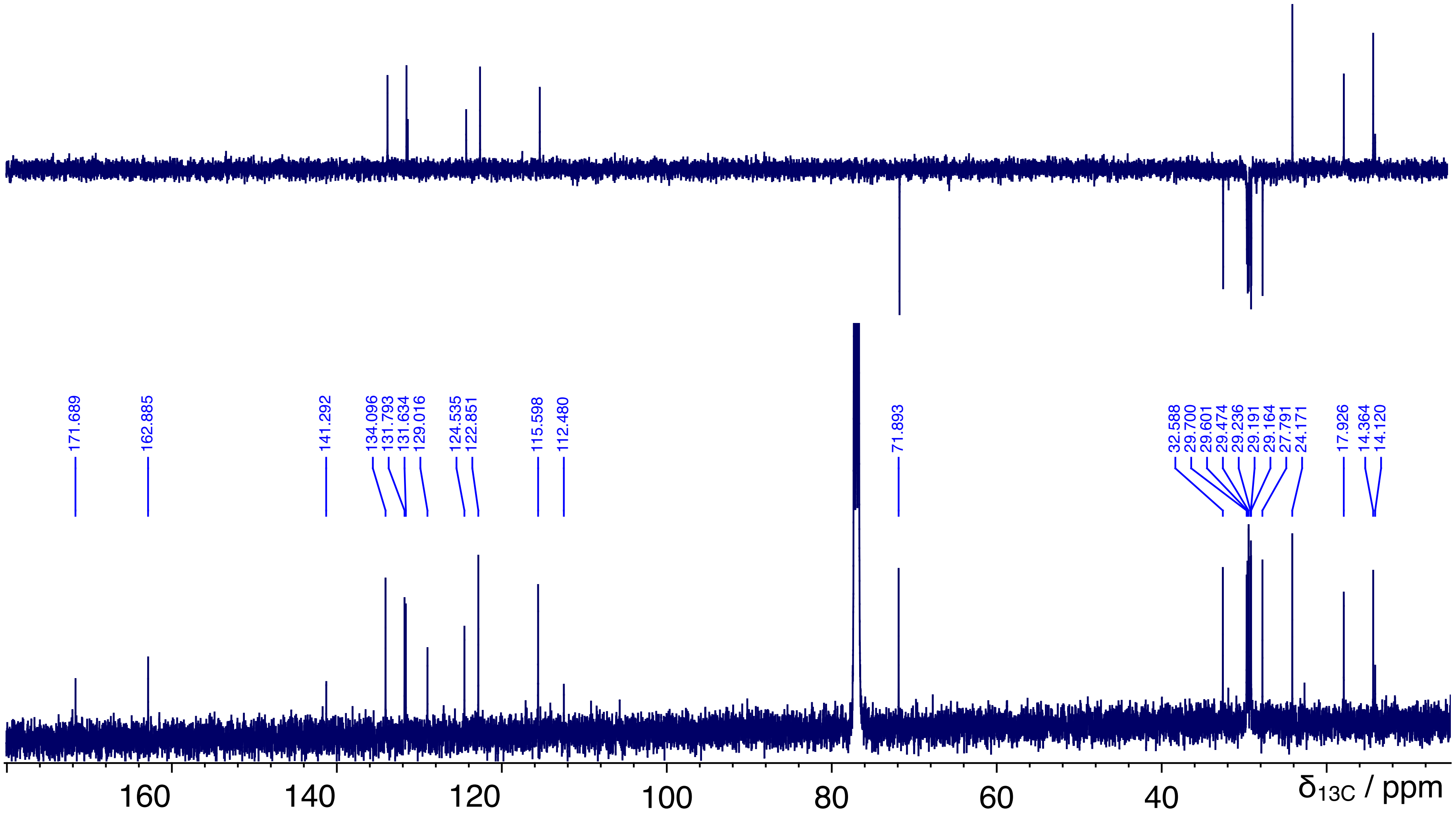

Figure S13. ^{13}C NMR and DEPT spectra ($CDCl_3$, 150 MHz) for **3**.

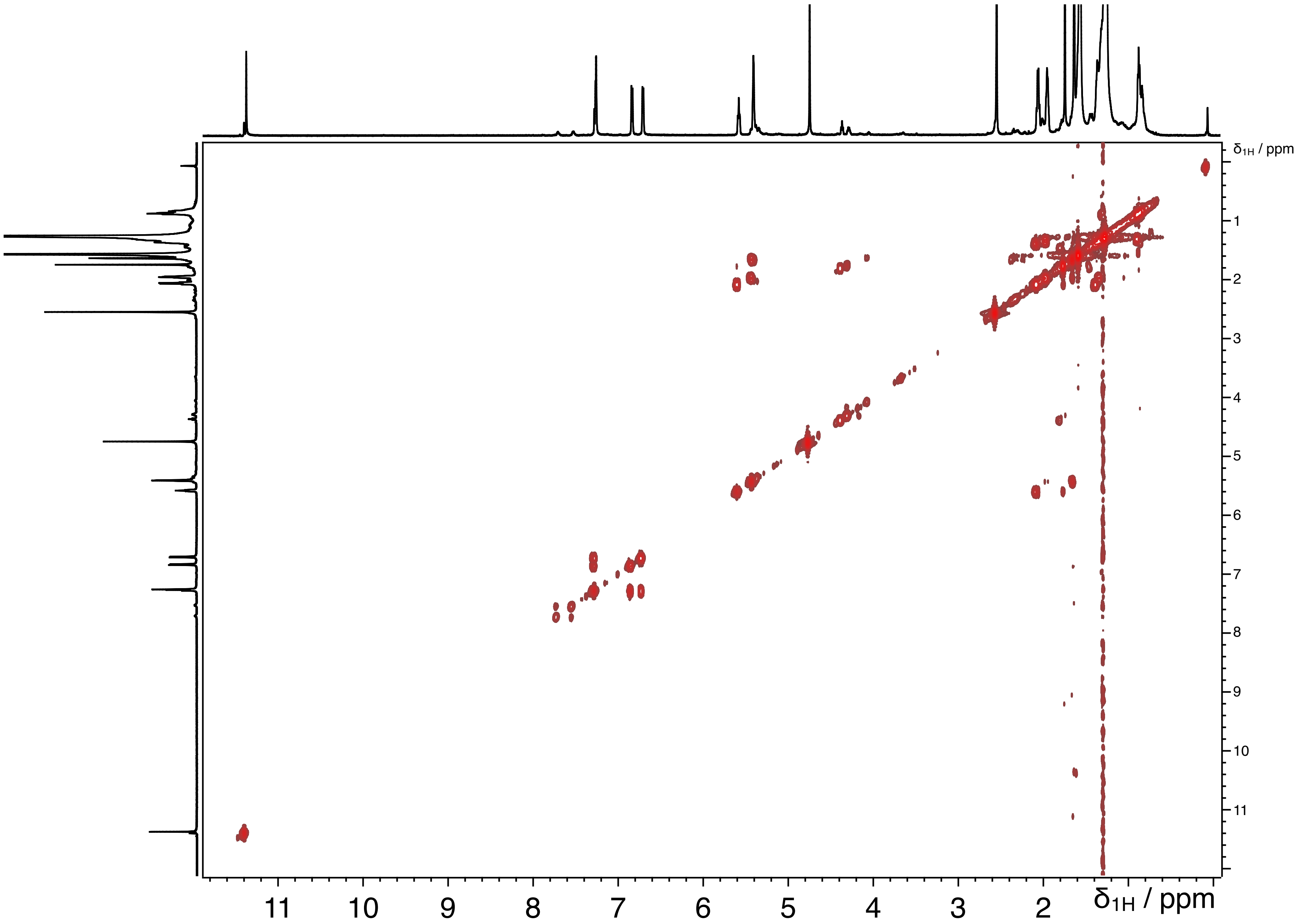

Figure S14. DQF-COSY spectrum ($CDCl_3$, 600 MHz) for **3**

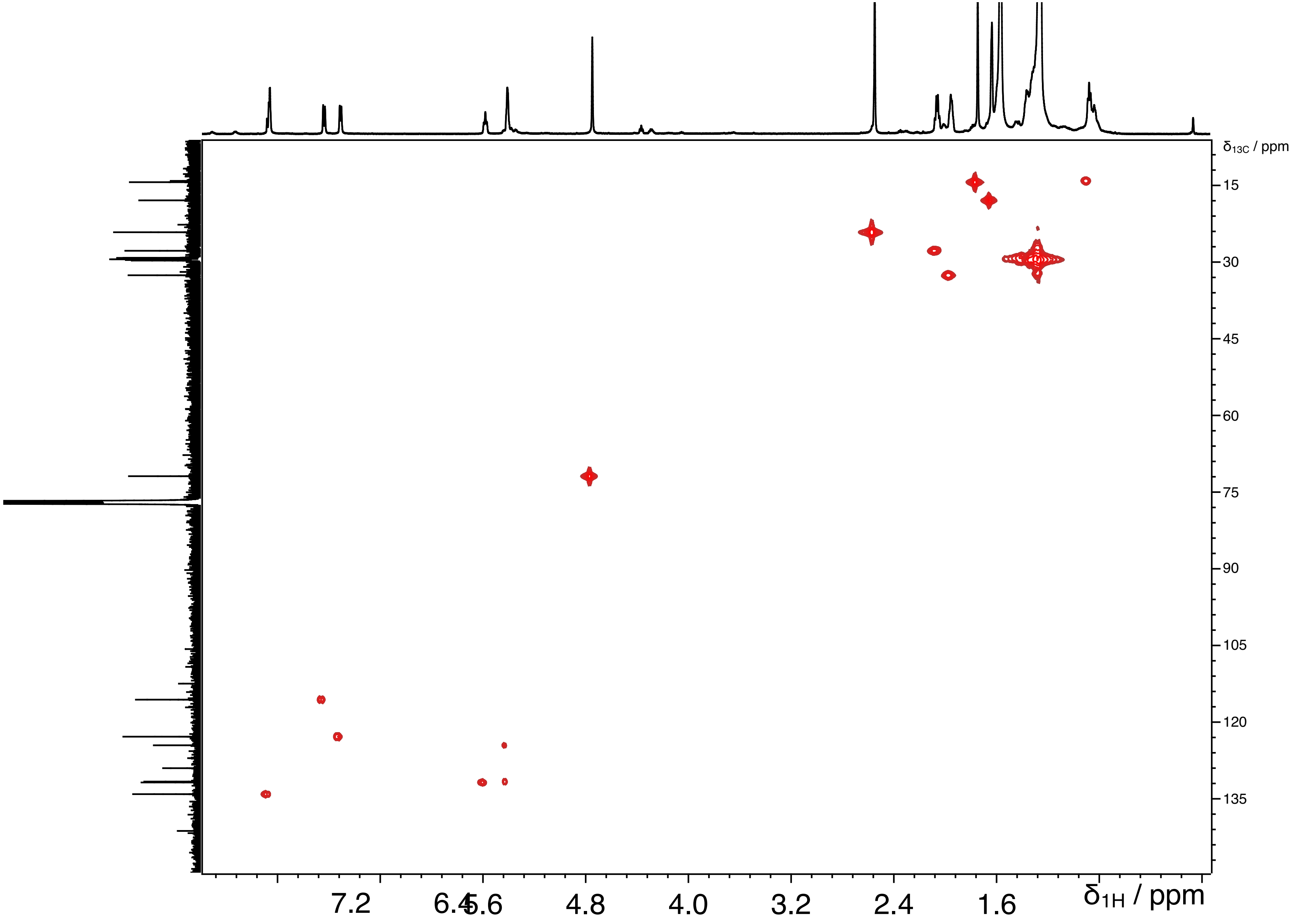

Figure S15. HMQC spectrum ($CDCl_3$, 600 MHz) for **3**.

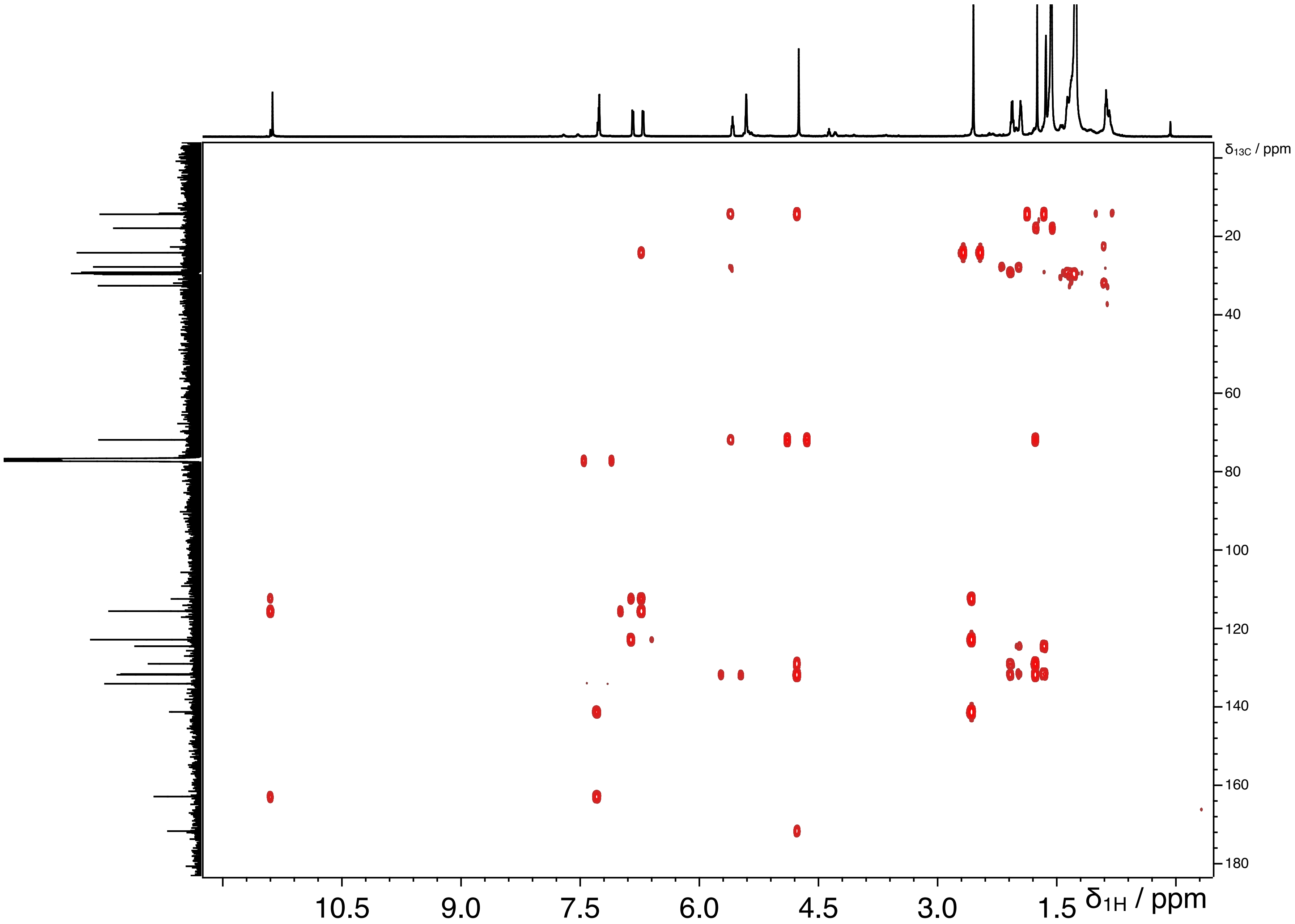

Figure S16. HMBC spectrum ($CDCl_3$, 600 MHz) for **3**.

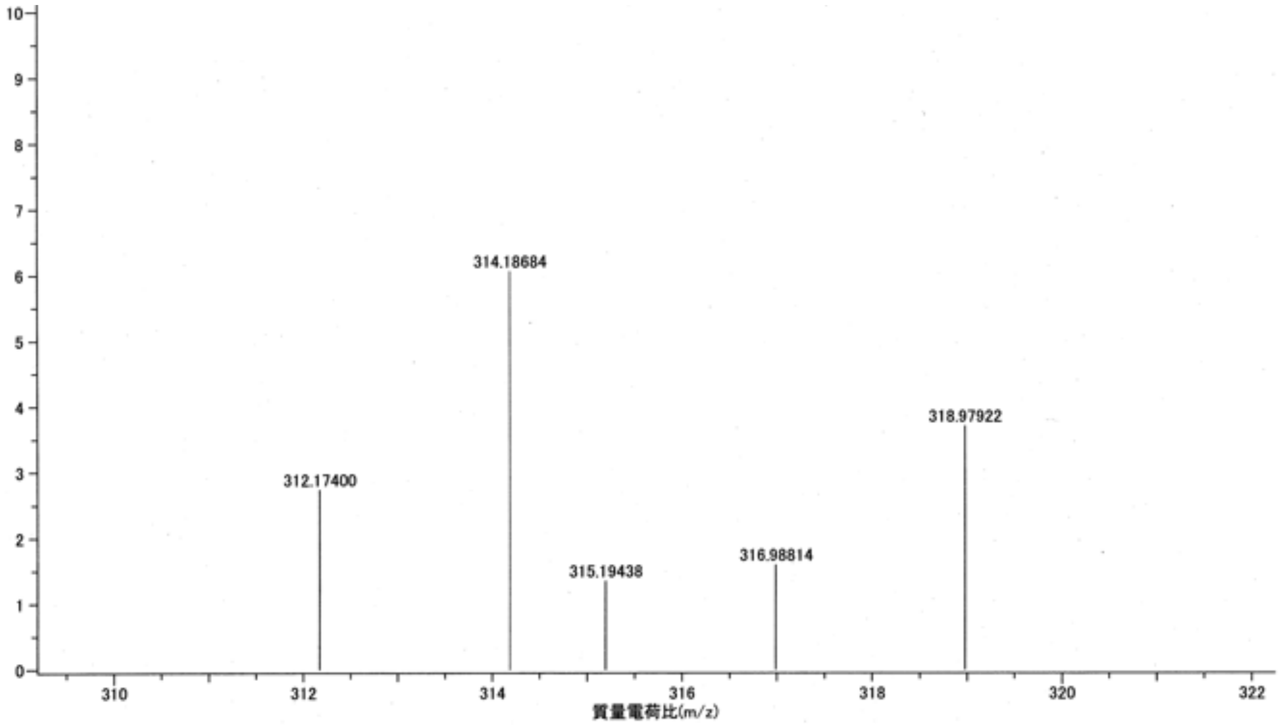

Figure S17. HRMS spectrum for **1**.

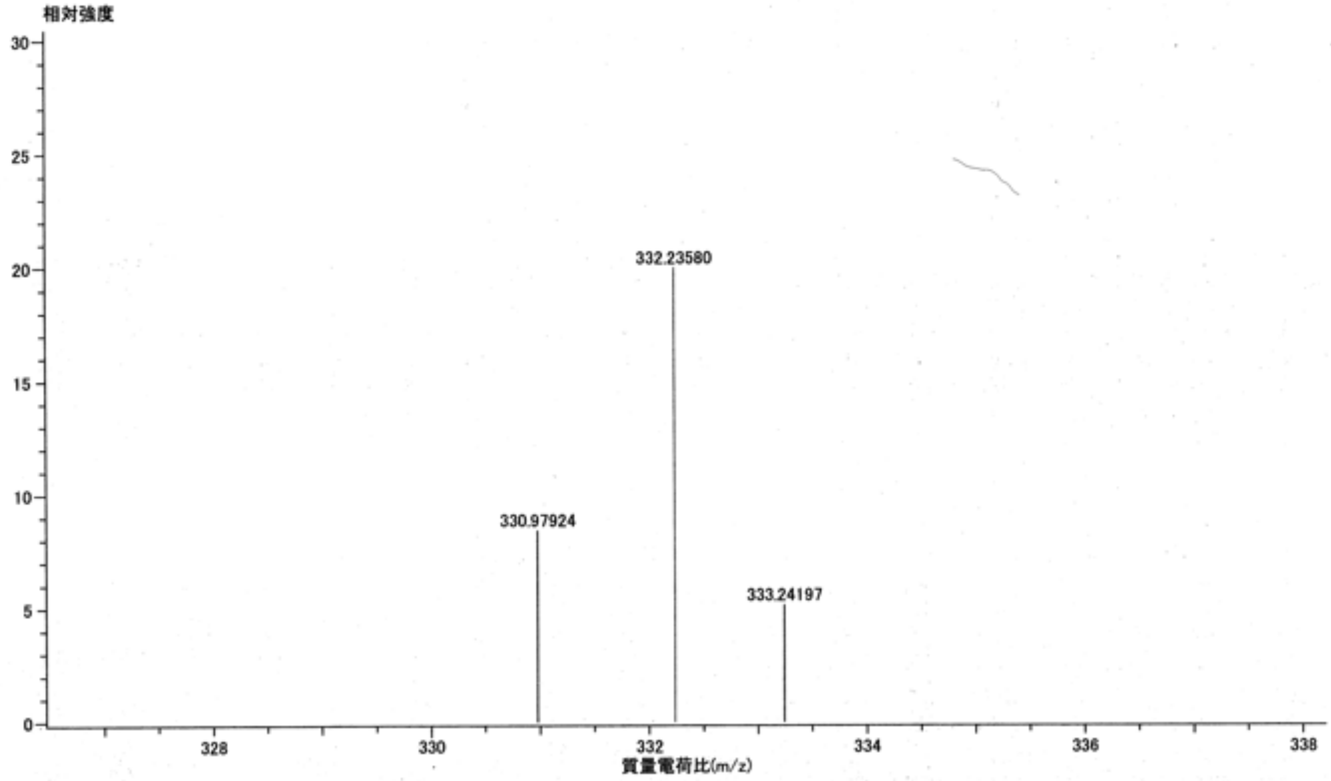

Figure S18. HRMS spectrum for **2**.

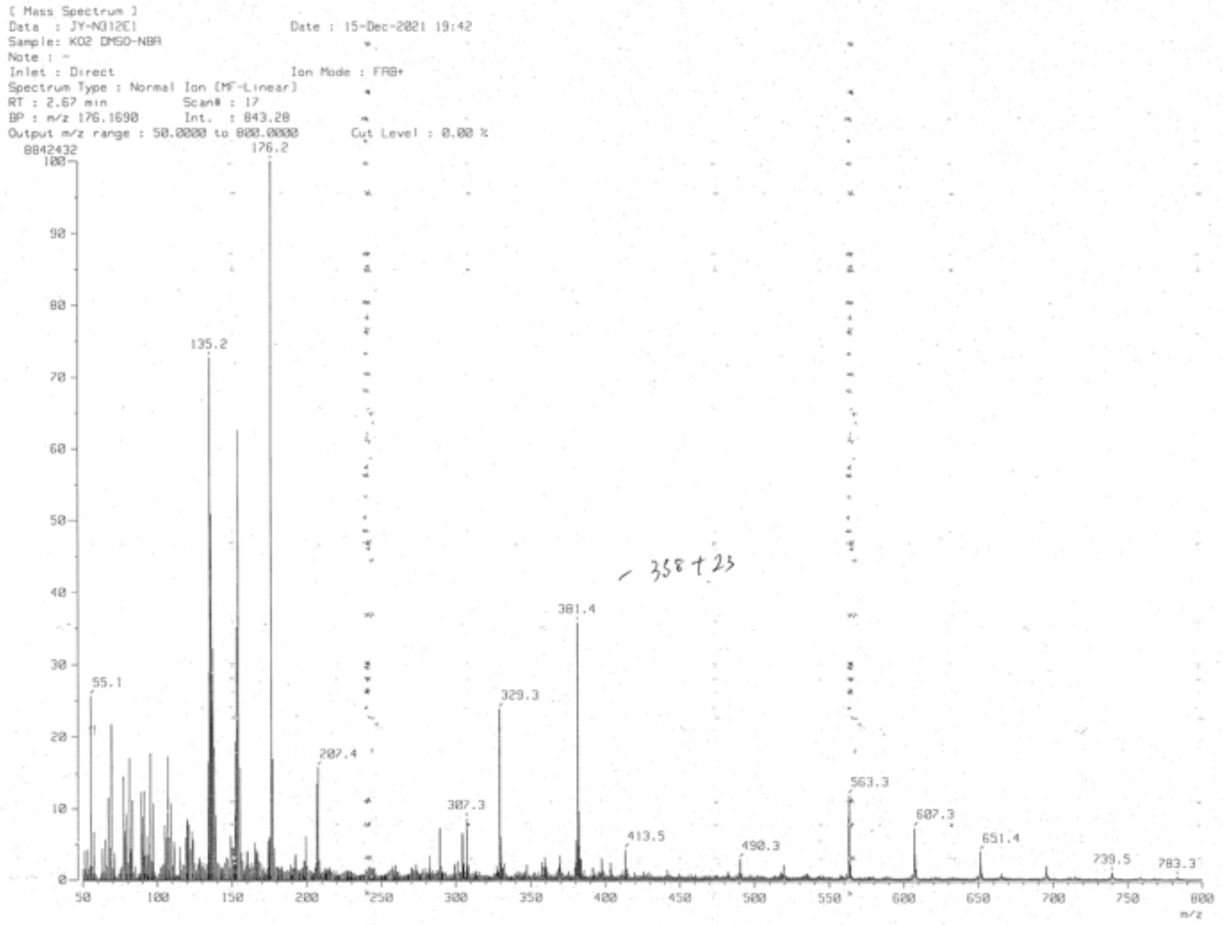


```
[ Elemental Composition ]                                    Page: 1
Data   : J028                          Date : 27-Dec-2021 21:22
Sample: N312-e1 DMSO-NBA
Note :
Inlet : Direct                          Ion Mode : FAB+
RT : 2.34 min                           Scan#: 15
Elements : C 30/15, 1H 45/25, O 5/0, Na 2/0
Mass Tolerance          : 20mmu
Unsaturation (U.S.)  : 0.0 - 100.0

Observed m/z Int%    Err[ppm / mmu]    U.S.  Composition
  381.2380   100.0     +42.5 / +16.2   14.5  C 28 1H 29 O
                       -12.9 /  -4.9    9.5  C 25 1H 33 O 3
                       -46.6 / -17.8   10.5  C 27 1H 34 Na
                       +48.8 / +18.6   11.5  C 26 1H 30 O Na
                        -6.6 /  -2.5    6.5  C 23 1H 34 O 3 Na       ←
                       -40.3 / -15.4    7.5  C 25 1H 35 Na 2
                        -0.3 /  -0.1    3.5  C 21 1H 35 O 3 Na 2
```

Figure S19. HRMS spectrum for **3**.

Reference

1. Choukchou-Braham, N.; Asakawa, Y.; Lepoittevin, J. Isolation, Structure Determination and Synthesis of New Dihydroiso-coumarins from Ginko biloba L. *Tetrahedron Lett.* **1994**, *35*, 3949–3952.
2. Li, W.; Wiesenfeldt, M.P.; Glorius, F. Ruthenium-NHC-Diamine Catalyzed Enantioselective Hydrogenation of Isocoumarins. *J. Am. Chem. Soc.* **2017**, *139*, 2585–2588.
